# Glucosinolate Content and Sensory Evaluation of Baby Leaf Rapeseed from Annual and Biennial White‐ and Yellow‐Flowering Cultivars with Repeated Harvesting in Two Seasons

**DOI:** 10.1111/1750-3841.14680

**Published:** 2019-06-25

**Authors:** Marie Groenbaek, Ulla Kidmose, Erik Tybirk, Hanne Lakkenborg Kristensen

**Affiliations:** ^1^ Dept. of Food Science, Faculty of Science and Technology Aarhus Univ. Kirstinebjergvej 10 Aarslev DK‐5792 Denmark; ^2^ Knold & Top ApS Fyrrevænget 1 Odder DK‐8300 Denmark

**Keywords:** baby leaf, bitterness, *Brassica napus* var. *oleifera*, glucosinolate, repeated harvesting, sensory evaluation

## Abstract

The chemical and sensory quality of field‐grown vegetables may be influenced by cultivar choice and agronomic factors but knowledge is lacking on the new rapeseed vegetables. White‐ and yellow‐flowering rapeseed cultivars were tested in two seasonally different field studies in Denmark at three different growing stages by early sowing the first year and late sowing the second year. Content of glucosinolates (GLSs) was analyzed, and the sensory quality of baby leaf samples was evaluated. The GLS content differed among cultivars across years in all growing stages, with biennial cultivars having the highest GLS content. In the second year, a higher content of all identified GLSs was found at two growing stages except for neoglucobrassicin and gluconasturtiin, compared to the first year. On the contrary, higher contents of all identified GLSs were found at a third stage in the first year except for progoitrin and 4‐methoxy glucobrassicin. Sensory evaluation of bitterness revealed differences among cultivars, higher intensities of bitterness in biennial cultivars, and a relationship between bitterness and content of bitter‐tasting and total GLSs. The effect of repeated harvesting on GLS content differed between the years and no general pattern was seen, except that the composition of individual GLSs was comparable for the biennial cultivars. We conclude that growing season and life cycle had a stronger influence on GLS content than stage at harvest. The link between bitter‐tasting GLSs and bitterness revealed that life cycle and seasonal effects affected the sensory profile of baby leaf rapeseed thereby making a healthier product due to high content of health‐beneficial GLSs.

## Introduction

Baby leaves from white‐flowering rapeseed (*Brassica napus* L. var. *oleifera*) are a relatively new crop in the Western world, as the trait white flower has only recently been reintroduced into modern cultivars of yellow‐flowering cultivars of rapeseed (Groenbaek, Tybirk & Kristensen, 2018a). These new cultivars have proved suitable for baby leaf production. In rapeseed, annual (spring) or biennial (winter) cultivars are available for cultivation. In Northern Europe, annual cultivars are sown in spring and harvested in late summer, whereas biennial cultivars are sown in late summer/early autumn and harvested the following summer. Therefore, different cultivars possess different qualities with respect to the need for vernalization and winter hardiness, among other things. Based on practical experience, baby leaf salad of white‐flowering rapeseed is thought to taste more mild and less bitter and astringent than baby leaf salad from yellow‐flowering rapeseed.

Over time, glucosinolates (GLSs) have been a major topic in rapeseed breeding and cultivation, as high levels of GLSs in the seeds can cause goitronic and other harmful effects in sensitive animals such as pigs, when the press cake is used as fodder (Bell, 1984). However, the negative effects have been markedly reduced due to low levels of GLSs in the seeds of modern cultivars. When rapeseed leaves are grown as a vegetable, the GLS‐related challenges are less relevant, as the estimated daily intake of GLSs by humans is low compared to the press cake intake when used as protein fodder (Steinbrecher & Linseisen, 2009; Woyengo, Beltranena, & Zijlstra, 2017). Other well‐known *Brassicas* grown as baby leaves are the kales *B. oleracea* var. *sabellica/acephala* and rape kale *B. napus* var. *pabularia*. Due to their content of GLSs and their breakdown products, the *Brassica* vegetables are thought to possess health‐beneficial properties such as reduced risk of cancer development and cardiovascular disease mortality (Manchali, Chidambara Murthy, & Patil, 2012; Zhang et al., 2011). The GLS breakdown products isothiocyantes, nitriles, and thiocyanates are formed after tissue disruption and are initially a defense mechanism evolved in the plant to avoid herbivory (Textor & Gershenzon, 2009).

In relation to optimizing GLS content for human health benefits, many studies have been made on the effect of plant age and developmental stage on GLS content. For example, an increase in the content of aliphatic GLSs was found in different kinds of kale throughout plant development (Groenbaek et al., 2016; Velasco, Cartea, Gonzalez, Vilar, & Ordas, 2007). Additionally, seasonal effects on GLS content due to different radiation and temperature levels have been exploited where effects of temperature, photosynthetic photon flux, and day length depended on the type and cultivar of different *B. oleracea* (Charron, Saxton, & Sams, 2005). The possibilities of repeated harvesting are of interest with regard to reducing the use of resources for vegetable growing in a more sustainable way (Kristensen & Stavridou, 2017). Even though repeated harvesting is common practice, at least in baby leaf rocket production, only few studies have been conducted (Hall, Jobling, & Rogers, 2015). Hall et al. (2015) found that the content of total GLSs decreased in a second harvest, as 4‐hydroxy glucobrassicin was not detected in the perennial wall rocket (*Diplotaxis tenuifolia* L. DC.) and glucobrassicin and 4‐methoxy glucobrassicin were absent in the second harvest of the annual garden rocket (*Eruca sativa* Mill.). They were all detected in the first harvest. Another study found only increasing content of primarily aliphatic GLSs in the same species due to several repeated harvests, arguing that due to the stress imposed on the plants, an elevated level of the defense compounds, in this case GLSs, was observed (Nitz & Schnitzler, 2002).

Some of the GLSs and their breakdown products may also have an effect on the sensory profile of baby leaf rapeseed, because they contribute to a bitter taste and high intensity of pungency in other vegetables. These GLSs include mainly sinigrin (2‐propenyl GLS), gluconapin (3‐butenyl GLS), progoitrin (2‐hydroxy‐3‐butenyl), glucobrassicin (3‐indolylmethyl), and neoglucobrassicin (1‐methoxy‐3‐indolylmethyl; Drewnowski & Gomez‐Carneros, 2000; Engel, Baty, le Corre, Souchon, & Martin, 2002; Mithen, Dekker, Verkerk, Rabot, & Johnson, 2000; Mølmann et al., 2015; Pasini, Verardo, Cerretani, Caboni, & D'Antuono, 2011; Schonhof, Krumbein, & Bruckner, 2004; van Doorn et al., 1998). However, so far no sensory evaluation of rapeseed baby leaves has been reported. Bitterness and pungency has previously been linked to reduced consumer acceptance (Drewnowski & Gomez‐Carneros, 2000), thereby introducing a dilemma between the positive health aspect of GLSs and the dislike of bitterness.

The objectives of the study were to investigate if cultivar selection of white and yellow‐flowering rapeseed cultivars, including qualities ascribed to annual and biennial cultivars, influenced the GLS content and composition as well as the sensory profile in baby leaf salad across two years. Furthermore, the objective was to test the effects of repeated harvesting and intact plants (grown unharvested for the same period) on GLS content and composition in a field experiment. The study was conducted with shifted sowing and harvesting dates in two consecutive years in order to address the influence of seasonal variation and rate of development on the phytochemicals and sensory properties.

## Materials and Methods

### Plant material

**Figure 1:**
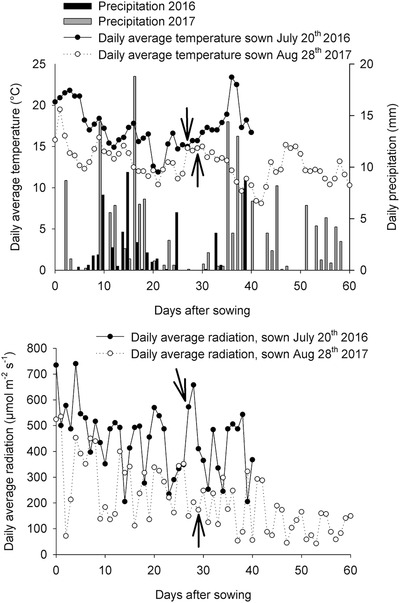
Climatic data from the two growing seasons, 2016 and 2017 showing daily average temperature (°C), daily precipitation (mm), and daily average radiation (µmol/m^2^/s). Arrows indicate baby leaf harvest (stage I), 27 (2016) and 29 (2017) days after sowing (DAS). End of data points indicate harvest at stage II (baby leaf re‐growth and intact plants) 40 and 60 DAS in 2016 and 2017, respectively.

The experiments were situated in the organic experimental fields of the Dept. of Food Science in Aarslev, Denmark (55°18′ N, 10°27′ E) on a sandy loam (Typic Agrudalf) containing 118 and 141 kg of mineral nitrogen (N) ha^−1^ and 1.1 and 0.7 kg of sulfur (S) ha^−1^ in the top 25‐cm soil layer in 2016 and 2017, respectively. We distributed 80 kg of N and 0.9 kg of S ha^−1^ in 2016 and 40 kg of N and 0.2 kg of S ha^−1^ in 2017 1 month before sowing in the form of dried chicken manure (4‐1‐3, DLG, Odense, Denmark). Additionally, 6 kg of S ha^−1^ were distributed in 2017 as kieserite (K+S Kali GmbH, Germany). Five cultivars of white‐flowering rapeseed (“Lysidé,” “SilverShadow,” “Jadak,” “Witt,” and “Lilput,” all from Knold & Top ApS, Odder, Denmark) and two cultivars of yellow‐flowering rapeseed (“Fenja” from NPZ‐Lembke, Holtsee, Germany and “Labrador” from KWS SAAT AG, Einbeck, Germany) were tested. They were sown early in 2016 on 20 July and late in 2017 on 28 August in plots of 1.6 × 5 m^2^ with 800 plants per m^2^ in 10 rows and organized in a completely randomized block design with three replications and extra plots outermost to eliminate border effects. An insect net (0.8 mm mesh size) covered the plants during the entire experiment. Maximum and minimum temperatures, global radiation, and precipitation were assessed by the Danish Meteorological Institute weather station situated in the experimental fields. The climatic data are shown in Figure 1.

At 27 and 29 days after sowing (DAS) in 2016 and 2017, respectively, the developmental stage of the baby leaf rapeseed (stage I) was recorded on the BBCH scale (Lancashire et al., 1991). We harvested 1 m^2^ of plants per plot just above the apical meristem using a hedge trimmer with a tray attached. The total weight of the harvested biomass was registered, and the leaves were stored in slightly perforated plastic bags for 2 days at 0.5 °C and 98% relative humidity until preparation for chemical and sensory analyses. Forty DAS (2016) and 60 DAS (2017) the intact plants (stage II_uncut_) were evaluated on the BBCH scale, and another square meter of intact plants was harvested per plot. Furthermore, a second cut (stage II_cut_), for example a repeated harvesting of the regrown leaves from the baby leaves harvested at stage I, was performed 40 and 60 DAS, respectively. The plants were stored as mentioned above.

### Chemical analyses

Approximately 150 g of fully unfolded leaves of different sizes with 2‐ to 3‐cm stalks were frozen in liquid N and freeze‐dried in a CHRIST freeze dryer, Gamma 1–20 (Osterode am Harz, Germany), from which the dry matter (DM) was calculated. Freeze‐dried plant material was grinded to 0.5 mm using a Retsch Mixer Mill MM 200 (SkanLab ApS, Slangerup, Denmark) and kept in the freezer (−24 °C) until further analysis.

GLS analysis were made in accordance with Beck, Jensen, Bjoern, and Kidmose (2014). In brief, 750 mg of sample material were extracted three times with 9, 6, and 6 mL of methanol (70%), respectively, using centrifugation and collection of the supernatant between extractions. The supernatant was adjusted to a total volume of 25 mL with methanol (70%), and 6 mL hereof were applied to a DEAE Sephadex A25 ion exchanger followed by a purified arylsulfatase solution and left for 16 hr. The desulfo compounds were eluted with 1 mL of purified water four times and the eluent filtered through a 0.45‐µm Q‐max nylon filter (Frisenette ApS, Knebel, Denmark). The samples were analyzed by high‐performance liquid chromatography (HPLC) on a Dionex Ultimate 3000 HPLC system (Germering, Germany). GLSs were identified by retention time of authentic standards (glucoiberin, sinigrin, progoitrin, glucoraphanin, gluconapin, glucobrassicanapin, glucobrassicin, 4‐hydroxyglucobrassicin, 4‐methoxyglucobrassicin, and neoglucobrassicin from C2 Bioengineering ApS [Karlslunde, Denmark]) and quantified by means of the internal standard, glucotropaeolin (PhytoLab, Vestenbergsgreuth, Germany).

### Sensory descriptive analysis

The sensory quality of the cultivars was measured in both 2016 and 2017. However, due to reduced growth and no harvest in the late sowing season, the cultivar “Lysidé” was not evaluated in 2017. The leaves of the harvested samples were washed carefully, centrifuged in a big cloth, and leaves with lamina of 5 to 6 cm and 3 to 5 cm in length were selected for sensory evaluation in both years. Stalks were snapped off at the length of 2 to 3 cm. In 2016, each evaluation sample consisted of two leaves from each cultivar field replicate (six leaves in total), and in 2017, each sample consisted of 4 g of leaves as a mixture of each cultivar field replicate.

The sensory evaluation was carried out as a sensory descriptive analysis using a trained sensory panel. The panel consisted of 11 assessors (10 females/1 male, aged 32 to 61 years) and 7 assessors (6 females/1 male, aged 26 to 61 years) in 2016 and 2017, respectively. In 2016, the assessors attended a 3‐hr discussion where they discussed and agreed on the following vocabulary of nine sensory aroma‐, flavor‐, taste‐, and mouth feeling descriptors based on samples of “Fenja,” “Witt,” and “Lilput”: flower vase water aroma, rapeseed aroma, fresh green aroma, horseradish aroma, sourness, bitterness, rapeseed flavor, peapod flavor, and astringent mouth feeling. Please consult Table S1 for attribute descriptions. During the discussion session, the sensory panel was introduced to reference samples of raw shredded horseradish, mustard, raw peapods, and raw radish.

**Table 1:** Glucosinolate (**µ**mol/g DM) content of seven white‐ or yellow‐flowering, annual or biennial rapeseed cultivars harvested as baby leaves (stage I) 27 and 29 days after sowing in 2016 and 2017, respectively

Year	Life cycle	Cultivar (cv)	Progoitrin	Glucobras‐sicanapin	Gluco‐brassicin	4‐Hydroxy glucobras‐sicin	4‐Methoxy glucobras‐sicin	Neogluco‐brassicin	Gluconas‐turtiin	Total indole GLSs	Total GLSs	Bitter‐tasting GLSs
2016	Annual	Lysidé (w)^a^	0.05 ± 0.00		2.73 ± 0.41	0.01 ± 0.01	0.33 ± 0.09	0.13 ± 0.04	0.01 ± 0.0	3.20 ± 0.54	3.26 ± 0.55	2.91 ± 0.44
		Silvershadow (w)	0.01 ± 0.00		1.45 ± 0.24	0.01 ± 0.01	0.41 ± 0.03	0.03 ± 0.01	0.00 ± 0.0	1.89 ± 0.23	1.90 ± 0.23	1.49 ± 0.25
		Fenja (y)	0.01 ± 0.01		2.54 ± 0.11	0.03 ± 0.01	0.51 ± 0.03	0.07 ± 0.0	0.03 ± 0.01	3.14 ± 0.12	3.18 ± 0.12	2.61 ± 0.11
	Biennial	Jadak (w)	0.04 ± 0.01		2.99 ± 0.26	0.05 ± 0.02	0.49 ± 0.02	0.10 ± 0.01	0.03 ± 0.01	3.64 ± 0.28	3.71 ± 0.29	3.13 ± 0.27
		Witt (w)	0.02 ± 0.01		3.22 ± 0.44	0.02 ± 0.02	0.75 ± 0.08	0.21 ± 0.03	0.02 ± 0.01	4.21 ± 0.55	4.26 ± 0.56	3.46 ± 0.48
		Lilput (w)	0.04 ± 0.01		3.08 ± 0.49	0.04 ± 0.03	0.58 ± 0.08	0.29 ± 0.07	0.03 ± 0.01	4.00 ± 0.67	4.07 ± 0.68	3.41 ± 0.56
		Labrador (y)	0.03 ± 0.01		2.98 ± 0.31	0.04 ± 0.02	0.54 ± 0.05	0.10 ± 0.01	0.01 ± 0.0	3.66 ± 0.33	3.70 ± 0.33	3.11 ± 0.32
Flower color effect			NS^b^		NS	NS	NS	NS	NS	NS	NS	NS
2017	Annual	Lysidé (w)										
		Silvershadow (w)	0.01 ± 0.00	0.01 ± 0.01	2.18 ± 0.08	0.02 ± 0.01	1.17 ± 0.08	0.01 ± 0.00	0.00 ± 0.01	3.40 ± 0.11	3.42 ± 0.11	2.21 ± 0.07
		Fenja (y)	0.01 ± 0.00	0.01 ± 0.00	3.97 ± 0.59	0.02 ± 0.02	1.06 ± 0.16	0.03 ± 0.00	0.21 ± 0.00	5.09 ± 0.72	5.35 ± 0.73	4.02 ± 0.59
	Biennial	Jadak (w)	0.05 ± 0.00	0.01 ± 0.00	5.09 ± 0.33	0.04 ± 0.00	1.01 ± 0.07	0.09 ± 0.01	0.03 ± 0.00	6.22 ± 0.39	6.34 ± 0.39	5.24 ± 0.34
		Witt (w)	0.05 ± 0.02	0.04 ± 0.01	4.29 ± 0.25	0.07 ± 0.01	1.38 ± 0.02	0.15 ± 0.02	0.03 ± 0.00	5.88 ± 0.27	6.00 ± 0.26	4.49 ± 0.24
		Lilput (w)	0.04 ± 0.00	0.02 ± 0.00	3.96 ± 0.29	0.02 ± 0.01	1.00 ± 0.08	0.15 ± 0.03	0.04 ± 0.01	5.13 ± 0.40	5.22 ± 0.39	4.15 ± 0.31
		Labrador (y)	0.03 ± 0.00	0.05 ± 0.02	4.51 ± 0.65	0.03 ± 0.01	1.18 ± 0.18	0.09 ± 0.01	0.01 ± 0.00	5.79 ± 0.83	5.88 ± 0.82	4.62 ± 0.65
Flower color effect			NS	NS	NS	NS	NS	NS	c	NS	NS	NS
Significance												
Cv			***	**	**	NS	NS	***	c	*	*	***
Life cycle			***	**	***	*	*	***	c	***	***	NS
Year			NS		***	NS	***	***	c	***	***	***
Life cycle * year			NS		NS	NS	NS	NS	c	NS	NS	NS

Values are means (*n* = 3) ± standard deviation. Cv significance obtained from *f*(*x*) = cv + block. Flower color effect obtained from *f*(*x*) = flower color + block within each year. Life cycle and year significance obtained from *f*(*x*) = life cycle + year + life cycle × year block.

^a^w, white flower color; y, yellow flower color.

^b^NS, not significant; ^*^, *P *≤ 0.05; ^**^, *P *< 0.01; ^***^, *P *< 0.001.

^c^Unable to perform statistics, as data could not be transformed to normal distribution or homogeneity of variance.

In addition, the sensory panel attended a 2‐hr training session each year prior to the sensory analysis. In the training session, the assessors evaluated the following four samples individually: “Fenja,” “Lilput,” “Witt,” and “Silvershadow” in three replicates in two blocks, whereas all the cultivars were evaluated in three replicates in three blocks during the test session with breaks in between. At both the training and test sessions, the samples were served in transparent plastic beakers with lids (ABENA A/S, Aabenraa, Denmark) labeled with a unique, three‐digit number in random order. The samples were kept at 15 °C until 1 hr before serving. A 15‐cm, nonstructured continuous scale with the anchors low intensity and high intensity, respectively, was used to rate the sensory descriptors, and the grades were registered electronically (Compusense cloud, sensory evaluation software). Training and descriptive analyses were performed in accordance with the international standard ISO 8586‐1 (ISO, 1993) and conducted in a sensory evaluation laboratory fulfilling the requirements set by the ASTM (1986).

### Statistical analysis

All statistical calculations were done in SAS (SAS Inst. Inc., Cary, NC, USA, release 9.4, 2000) using a general linear model to test least squares means separations (*P* ≤ 0.05) of GLS content effects and sensory attributes effects from cultivars, life cycle (annual/biennial), year, and life cycle × year. The mixed model procedure was used to test least squares means separations (*P* ≤ 0.05) of GLS content effects from cultivar, growing stage, and cultivar × growing stage, with growing stage being a repeated variable, as harvest was performed on the same plants and in the same plot twice. When needed, data were transformed in order to reach normal distribution and homogeneity of variance.

PanelCheck v. 1.4.2. (www.Panelcheck) was used to give the sensory panel assessors feedback. Unscrambler X10.5.1. (Camo software, Oslo, Norway) was used as software for multivariate statistical analysis of sensory and chemical data using principal component analysis (PCA).

## Results and Discussion

### Effect of growing stage on glucosinolate content and composition

The following GLSs were identified from baby leaves in all stages: the aliphatic GLSs progoitrin ((R)‐2‐hydroxy‐3‐butenyl GLS), glucobrassicanapin (4‐pentenyl GLS), and glucoraphanin (4‐methylsulphinylbutyl GLS). Glucobrassicanapin and glucoraphanin were only identified in 2017 and glucoraphanin only in traces, for which reason no data is presented. The indole GLSs glucobrassicin (3‐indolymethyl GLS), 4‐hydroxy glucobrassicin (4‐hydroxy‐3‐indolylmethyl GLS), 4‐methoxy glucobrassicin (4‐methoxy‐3‐indolylmethyl GLS), and neoglucobrassicin (1‐methoxy‐3‐indolylmethyl GLS), and the aromatic gluconasturtiin (2‐phenylethyl GLS). Bitter‐tasting GLSs is the sum of progoitrin, glucobrassicin, and neoglucobrassicin. Total GLSs is the sum of all identified GLSs.

**Figure 2:**
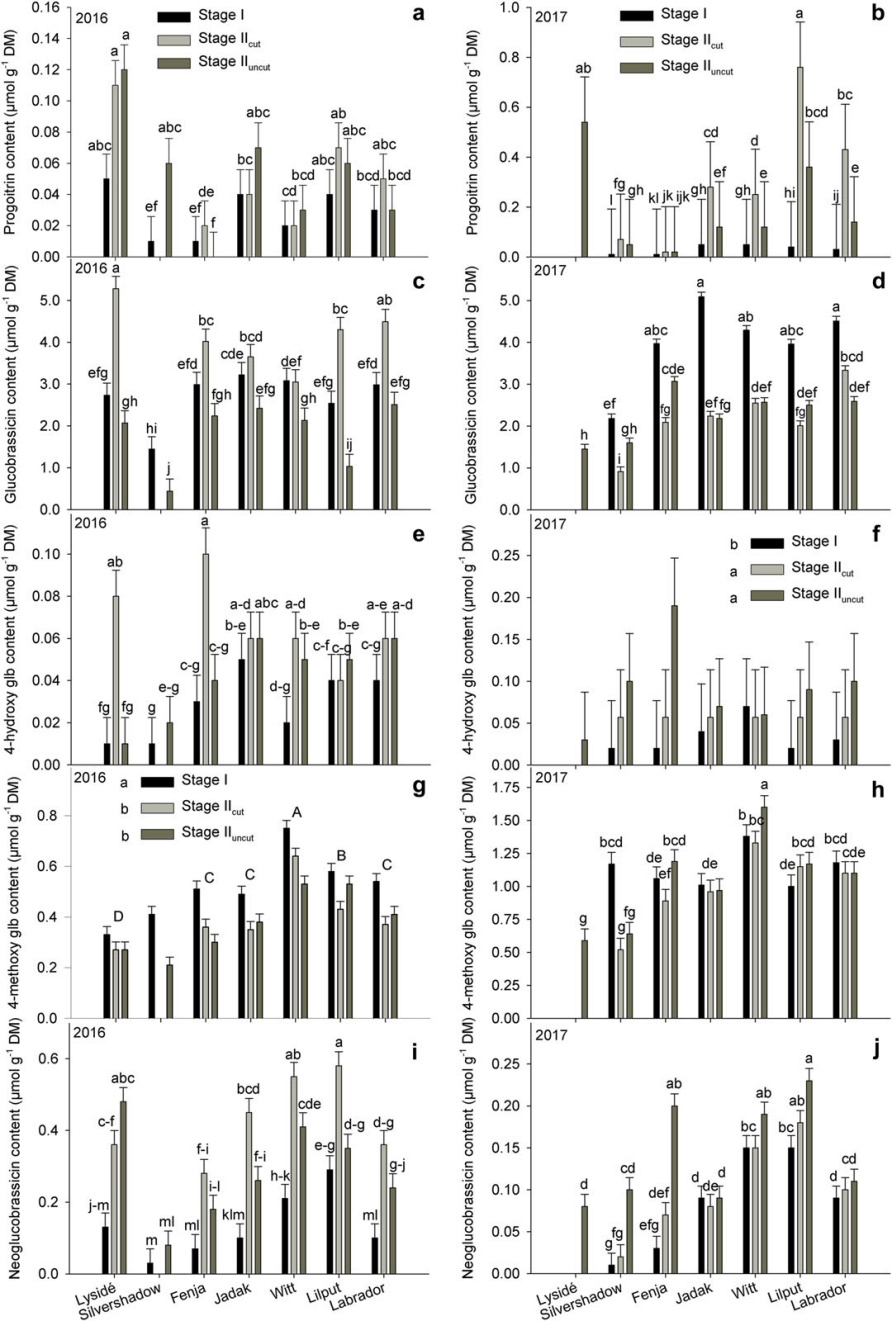
Content of glucosinolates (µmol/g DM) in seven white‐ or yellow‐flowering cultivars of rapeseed at the growing stages baby leaf, stage I (black bars), baby leaf re‐growth, stage II_cut_ (light grey bars), and intact plants, stage II_uncut_ (dark grey bars). Note the different scales of *y*‐axes to allow direct comparison of responses. Different letters indicate significant differences (*P* ≤ 0.05) among growing stages (f, small letters) and cultivars (g, capital letters) or cultivar × growing stage (a). When no interaction was found, “Silvershadow” and “Lysidé” were excluded from the statistical calculations due to missing values. Glb, glucobrassicin. Bars represent standard errors.

**Figure 3:**
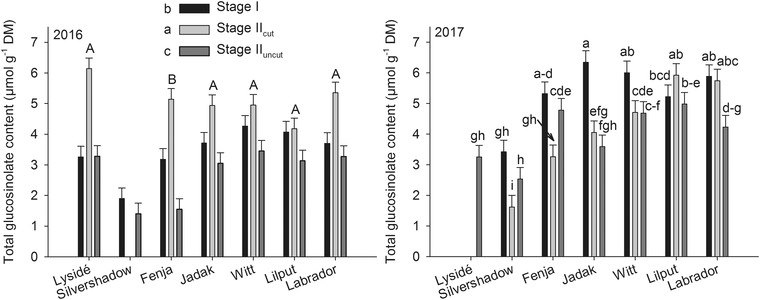
Content of total glucosinolates (µmol/g DM) in seven white‐ or yellow‐flowering cultivars of rapeseed at the growing stages baby leaf, stage I (black bars), baby leaf re‐growth, stage II_cut_ (light grey bars), and intact plants, stage II_uncut_ (dark grey bars). Different letters indicate significant differences (*P* ≤ 0.05) among cultivars (2016, capital letters) and growing stages (2016, small letters) or cultivar × growing stage (2017). When no interaction was found, “Silvershadow” and “Lysidé” were excluded from the statistical calculations due to missing values. Bars represent standard errors.

The effects of growing stage on the identified GLSs differed, and an interaction between cultivar and growing stage was found for all identified GLSs except for 4‐methoxy glucobrassicin and gluconasturtiin (data not shown) in 2016 and 4‐hydroxy glucobrassicin in 2017 (Figure 2). The plants harvested at stage II_cut_ had the highest content of progoitrin for all the biennial cultivars (“Jadak,” “Witt,” “Lilput,” and “Labrador”) in 2017, whereas differences between growing stages were only seen for “Fenja” in 2016. For the indole GLS group, effects of growing stage differed between the years, as stage II_cut_ contained the highest levels of glucobrassicin, neoglucobrassicin, and 4‐hydroxy glucobrassicin for the annual cultivars (“Lysidé” and “Fenja”) in 2016. In 2016, the content of total GLSs was higher at stage II_cut_ than at stage I, which was again higher than stage II_uncut_ when evaluated as mean values of cultivars (Figure 3). In 2017, the picture was quite different, and for most cultivars stage I plants had a higher content of total GLSs than the other two growing stages, or at least one of them.

In a study on the Portuguese Couve‐nabica (*B. napus*), the content of total GLSs increased from sowing till 8 weeks after sowing, corresponding to stage II_uncut_ in this study (harvested approximately 6 and 9 weeks after sowing; Rosa, Heaney, Portas, & Fenwick, 1996) The results of the study by Rosa et al. (1996) differed from our results, though. However, looking only at the indole GLS contents of the Couve‐nabica, a decrease was found in the same period. This result is comparable to our findings, as the content of the most abundant indole GLSs, glucobrassicins, and 4‐methoxy glucobrassicin at stage II_uncut_ was lower than at stages I and II_cut_. Furthermore, a negative linear relationship between glucobrassicin (*P* < 0.0001, *r* ^2^ = 0.28) and 4‐methoxy glucobrassicin (*P* = 0.0002, *r* ^2^ = 0.16) and the BBCH scale code was found. In general, the share of indole GLSs compared to aliphatic GLSs was very high in the tested rapeseed cultivars compared to other *Brassicas* (Bhandari, Jo, & Lee, 2015; Hall et al., 2015), and in accordance with the Couve‐nabica (Rosa et al., 1996) and the winter and spring rapeseed cultivars tested by Sarwar and Kirkegaard (1998). This trend was confirmed in sprouts and seedlings of the tested cultivars from this study and could very well be a result of the double low rapeseed characteristics, which both the tested cultivars and canola possess (Groenbaek, Tybirk, & Kristensen, 2018b). The responses in GLS content to the different growing stages were not the same in 2016 and 2017. In 2016, the annual cultivars were developing toward flowering (intact plants/stage II_uncut_), whereas the biennial cultivars only faced vernalization in 2017. This could cause different responses in the GLS biosynthesis, as developmental stage has previously been found to alter GLS content and composition in rapeseed (Clossais‐Besnard & Larher, 1991) and hence affect the sensory properties. However, as only the annual cultivars in 2016 underwent the transition from vegetative stage to flowering stage, the growing conditions differing due to seasonal variation between the years must have been the main driver for differences in GLS content and composition. The divergent responses among the annual cultivars with respect to progoitrin and neoglucobrassicin content (Figures 2a, b, and i) could be explained by their difference in genetic origin, as “Lysidé,” “Silvershadow,” and “Fenja” are not closely related except for the white flower character in “Lysidé” and “Silvershadow,” which originated from the cultivar “Hobson.” In contrast, the biennial cultivars were rather closely related, as they contained at least 62% similar genes due to backcrossing to “Labrador.” The flower color of “Jadak,” “Witt,” and “Lilput” originates from the rapeseed cultivar “Bianca” (Tybirk, personal communication).

The effect of repeated harvesting on the GLS content of baby leaf salad has only been investigated to a limited degree (Hall et al., 2015; Nitz & Schnitzler, 2002). White‐flowering rapeseed is a new salad crop, and only one study of growth and phytochemical content in the context of baby leaf production have been published to date (Groenbaek et al., 2019). Although we found differing effects of repeated harvesting, Hall et al. (2015) consequently experienced a reduced content of total GLSs in the second harvest independently of the species and season in perennial wall rocket and annual garden rocket. This was mainly due to the lack of identification of the indole GLSs in the second harvest, which led to the decrease.

An increase of GLSs at stage II_cut_ could be expected, as it mimics an abiotic stress such as herbivory or grazing (Textor & Gershenzon, 2009). This theory was supported by the 2016 results, but not by the results from 2017. This could be explained by relatively high average temperatures (18.0 °C in 2016 and 12.1 °C in 2017), less precipitation (13.2 mm in 2016 and 87.3 mm in 2017), and higher average daily radiation (411 µmol/m^2^/s in 2016 and 146 in µmol/m^2^/s 2017) in 2016, compared to 2017, during the period from the harvest at stage I till harvest of stage II (Figure 1). This may have led to conditions of induced stress, resulting in a higher content of GLSs adding to the stress of cutting. In general, higher temperatures have been linked to increased GLS content (Neugart et al., 2018). For the indole GLSs, this could be due to a temperature effect on the indole‐3‐acetic pathway, where higher temperatures favor an indole‐3‐acetic biosynthesis without tryptophan, leading to a higher pool of tryptophan available for indole GLS biosynthesis, as argued by Charron et al. (2005). As glucobrassicin is the precursor of 4‐methoxy glucobrassicin, 4‐hydroxy glucobrassicin, and neoglucobrassicin, the elevated temperatures in 2016 compared to 2017 might also have inhibited methoxylation, which Zhang et al. (2008) suggested was the case for turnip root (*B. rapa* ssp. *rapifera* L.). Indeed, a share of 80% of glucobrassicin of the total GLS content at stage II_cut_ from 2016 indicates an accumulation.

### Effect of cultivar, life cycle, and year on glucosinolate content and composition

Across years, cultivar effects were found in four out of seven GLSs, the total indole GLSs and total GLSs at stage I (Table 1). An effect of life cycle was found for all individual GLSs and the total content of GLSs, where the biennial cultivars in general had a higher content of GLSs than the annual cultivars. In contrast, in a previous study the annual cultivar “Silvershadow” had a higher content of all identified indole GLSs as well as total GLSs than biennial cultivars, when the same cultivars were grown as sprouts and seedlings (Groenbaek et al., 2018a).

At stage I, differences in total GLS content between the 2 years were quite distinct and were evaluated to be 57% higher for the annual cultivars and 48% higher for the biennial in 2017 compared to 2016. This difference was mainly due to elevated levels of glucobrassicin and 4‐methoxy glucobrassicin in 2017 compared to 2016, whereas the content of neoglucobrassicin was higher in 2016.

The differences between years in terms of GLS content could be due to a relationship between plant yield and the fact that a dilution effect can occur, as the plants grow bigger (Groenbaek & Kristensen, 2014). In our case, the yield was approximately twice as high in 2016 compared to 2017 (Groenbaek et al., 2019). This was supported by a negative linear relationship at stage I between yield and total GLS content with both years included (*P* = 0.0009, *r* ^2^ = 0.26). In addition, a negative linear relationship between total GLSs and BBCH scale code (data not shown) for stages I and II_uncut_ was found (*P* < 0.0001, *r* ^2^ = 0.24), supporting this theory. Furthermore, a higher level of S in the soil in 2017 could also have contributed to elevated GLS levels, as described by Groenbaek et al. (2014) in a study on field‐grown curly kale.

Cultivar and life cycle effects were also found at stage II_uncut_ and stage II_cut_, but to a lesser extent than at stage I (Table 2 and 3). Life cycle effects were due to a generally higher content of GLSs in the biennial cultivars, compared to the annual ones, as found at stage I. An effect of year occurred frequently at stages II_uncut_ and II_cut_, but different GLSs were affected when compared to stage I. The content of all identified GLSs, except for neoglucobrassicin, was higher in 2017 than in 2016 at stage II_uncut_, whereas only progoitrin and 4‐methoxy glucobrassicin were found in higher contents in 2017 than in 2016 at stage II_cut_. Furthermore, interactions between year and life cycle occurred at stage II_cut_, which could be explained by the lacking data from “Silvershadow” in 2016 and “Lysidé” in 2017, as the mean GLS content of the annual cultivars then was either higher (in 2016) or lower (in 2017) than it probably would have been, if the data had been present.

**Table 2:** Glucosinolate content (**µ**mol/g DM) of seven white‐ or yellow‐flowering, annual or biennial rapeseed cultivars harvested as intact plants (stage II_uncut_) 40 and 60 days after sowing in 2016 and 2017, respectively

Year	Life cycle	Cultivar (cv)	Progoitrin	Glucobras‐sicanapin	Gluco‐brassicin	4‐hydroxy glucobrassicin	4‐methoxy glucobrassicin	Neo‐glucobrassicin	Gluconas‐turtiin	Total indole GLSs	Total GLSs
2016	Annual	Lysidé (w)^a^	0.12 ± 0.02		2.07 ± 0.54	0.01 ± 0.01	0.27 ± 0.01	0.48 ± 0.14	0.33 ± 0.56	2.83 ± 0.68	3.28 ± 0.91
		Silvershadow (w)	0.06 ± 0.09		0.44 ± 0.16	0.02 ± 0.03	0.21 ± 0.05	0.08 ± 0.02	0.59 ± 0.52	0.76 ± 0.20	1.40 ± 0.38
		Fenja (y)	0.00 ± 0.00		1.03 ± 0.24	0.04 ± 0.01	0.30 ± 0.04	0.18 ± 0.01	0.01 ± 0.00	1.54 ± 0.27	1.55 ± 0.27
	Biennial	Jadak (w)	0.07 ± 0.05		2.24 ± 0.36	0.06 ± 0.05	0.38 ± 0.02	0.26 ± 0.06	0.03 ± 0.01	2.95 ± 0.46	3.05 ± 0.45
		Witt (w)	0.03 ± 0.01		2.42 ± 0.31	0.05 ± 0.02	0.53 ± 0.09	0.41 ± 0.07	0.01 ± 0.00	3.41 ± 0.38	3.45 ± 0.38
		Lilput (w)	0.06 ± 0.03		2.13 ± 0.41	0.05 ± 0.03	0.53 ± 0.10	0.35 ± 0.09	0.02 ± 0.01	3.06 ± 0.50	3.13 ± 0.55
		Labrador (y)	0.03 ± 0.01		2.51 ± 0.26	0.06 ± 0.04	0.41 ± 0.03	0.24 ± 0.02	0.01 ± 0.00	3.23 ± 0.31	3.27 ± 0.30
Flower color effect			NS^b^		NS	NS	NS	NS	NS	NS	NS
2017	Annual	Lysidé (w)	0.54 ± 0.03	0.51 ± 0.13	1.45 ± 0.52	0.03 ± 0.02	0.59 ± 0.15	0.08 ± 0.03	0.06 ± 0.01	2.15 ± 0.67	2.74 ± 0.68
		Silvershadow (w)	0.05 ± 0.01	0.01 ± 0.01	1.60 ± 0.35	0.10 ± 0.01	0.64 ± 0.04	0.10 ± 0.08	0.02 ± 0.01	2.44 ± 0.49	2.51 ± 0.47
		Fenja (y)	0.02 ± 0.01	0.01 ± 0.01	3.07 ± 0.78	0.19 ± 0.10	1.19 ± 0.18	0.20 ± 0.04	0.10 ± 0.02	4.64 ± 0.90	4.77 ± 0.89
	Biennial	Jadak (w)	0.12 ± 0.07	0.09 ± 0.07	2.18 ± 0.72	0.07 ± 0.08	0.97 ± 0.30	0.09 ± 0.07	0.05 ± 0.04	3.32 ± 1.02	3.50 ± 1.11
		Witt (w)	0.12 ± 0.04	0.08 ± 0.04	2.57 ± 0.32	0.06 ± 0.09	1.60 ± 0.10	0.19 ± 0.03	0.06 ± 0.01	4.42 ± 0.41	4.60 ± 0.40
		Lilput (w)	0.36 ± 0.10	0.53 ± 0.15	2.50 ± 0.32	0.09 ± 0.02	1.17 ± 0.06	0.23 ± 0.01	0.11 ± 0.00	3.98 ± 0.37	4.45 ± 0.39
		Labrador (y)	0.14 ± 0.03	0.12 ± 0.05	2.59 ± 0.76	0.10 ± 0.04	1.10 ± 0.30	0.11 ± 0.02	0.07 ± 0.01	3.90 ± 1.02	4.10 ± 0.98
Flower color effect			NS	NS	NS	NS	NS	NS	NS	NS	NS
Significance											
Cv			***	***	*	NS	NS	NS	NS	*	NS
Life cycle			NS	NS	***	NS	***	NS	NS	***	**
Year			***		*	**	***	**	NS	***	***
Life cycle * year			NS		NS	NS	NS	NS	NS	NS	NS

Values are means (*n* = 3) ± standard deviation. Cv significance obtained from *f*(*x*) = cv + block. Flower color effect obtained from *f*(*x*) = flower color + block within each year. Life cycle and year significance obtained from *f*(*x*) = life cycle + year life + cycle × year block.

^a^w, white flower color; y, yellow flower color.

^b^NS, not significant. ^*^, *P* ≤ 0.05;^**^, *P* < 0.01; ^***^, *P* < 0.001.

**Table 3:** Glucosinolate content (**µ**mol/g DM) of seven white‐ or yellow‐flowering, annual or biennial rapeseed cultivars harvested as baby leaf re‐growth (stage II_cut_) 40 and 60 days after sowing in 2016 and 2017, respectively

Year	Life cycle	Cultivar (cv)	Progoitrin	Glucobras‐sicanapin	Gluco‐brassicin	4‐hydroxy glucobrassicin	4‐methoxy glucobrassicin	Neo‐glucobrassicin	Gluconas‐turtiin	Total indole GLSs	Total GLSs
2016	Annual	Lysidé (w)^a^	0.11 ± 0.04		5.28 ± 2.04	0.08 ± 0.02	0.27 ± 0.04	0.36 ± 0.10	0.02 ± 0.01	6.00 ± 2.17	6.14 ± 2.14
		Silvershadow (w)									
		Fenja (y)	0.02 ± 0.00		4.30 ± 0.91	0.10 ± 0.01	0.36 ± 0.01	0.28 ± 0.08	0.07 ± 0.01	5.05 ± 1.01	5.14 ± 1.01
	Biennial	Jadak (w)	0.04 ± 0.03		4.02 ± 0.19	0.06 ± 0.02	0.35 ± 0.04	0.45 ± 0.08	0.02 ± 0.00	4.88 ± 0.21	4.94 ± 0.20
		Witt (w)	0.02 ± 0.01		3.65 ± 0.49	0.06 ± 0.03	0.64 ± 0.03	0.55 ± 0.15	0.02 ± 0.01	4.91 ± 0.66	4.95 ± 0.66
		Lilput (w)	0.07 ± 0.01		3.05 ± 0.21	0.04 ± 0.01	0.43 ± 0.05	0.58 ± 0.09	0.02 ± 0.00	4.10 ± 0.31	4.18 ± 0.31
		Labrador (y)	0.05 ± 0.02		4.49 ± 0.51	0.06 ± 0.03	0.37 ± 0.03	0.36 ± 0.05	0.02 ± 0.01	5.28 ± 0.51	5.35 ± 0.53
Flower color effect			NS^b^		NS	NS	NS	NS	NS	NS	NS
											
2017	Annual	Lysidé (w)									
		Silvershadow (w)	0.07 ± 0.03	0.03 ± 0.02	0.91 ± 0.06	0.05 ± 0.04	0.52 ± 0.07	0.02 ± 0.00	0.02 ± 0.01	1.50 ± 0.04	1.59 ± 0.07
		Fenja (y)	0.02 ± 0.01	0.02 ± 0.01	2.09 ± 0.30	0.08 ± 0.10	0.89 ± 0.12	0.07 ± 0.02	0.09 ± 0.00	3.13 ± 0.47	3.24 ± 0.48
	Biennial	Jadak (w)	0.28 ± 0.08	0.34 ± 0.10	2.24 ± 0.06	0.08 ± 0.05	0.96 ± 0.08	0.08 ± 0.01	0.07 ± 0.02	3.36 ± 0.06	3.71 ± 0.11
		Witt (w)	0.25 ± 0.05	0.29 ± 0.08	2.55 ± 0.27	0.07 ± 0.05	1.33 ± 0.12	0.15 ± 0.02	0.07 ± 0.01	4.10 ± 0.41	4.42 ± 0.41
		Lilput (w)	0.76 ± 0.07	1.60 ± 0.14	2.01 ± 0.41	0.07 ± 0.05	1.15 ± 0.09	0.18 ± 0.04	0.13 ± 0.03	3.42 ± 0.59	4.31 ± 0.67
		Labrador (y)	0.43 ± 0.03	0.55 ± 0.02	3.33 ± 0.85	0.14 ± 0.03	1.10 ± 0.21	0.10 ± 0.01	0.09 ± 0.01	4.67 ± 1.07	5.19 ± 1.10
Flower color effect			NS	NS	NS	NS	NS	NS	NS	NS	NS
Significance											
Cv			NS	***	**	NS	NS	NS	c	***	***
Life cycle			***	***	NS	NS	***	***	c	NS	NS
Year			***		***	NS	***	***	c	***	***
Life cycle * year			***		**	NS	NS	NS	c	**	***

Values are means (*n* = 3) ±standard deviation. Cv significance obtained from *f*(*x*) = cv block. Flower color effect obtained from *f*(*x*) = flower color + block within each year. Life cycle and year significance obtained from *f*(*x*) = life cycle + year + life cycle × year block.

^a^w, white flower color; y, yellow flower color.

^b^NS, not significant; ^*^, *P* ≤ 0.05; ^**^, *P* < 0.01; ^***^, *P* < 0.001.

^c^Unable to perform statistics, as data could not be transformed to normal distribution or homogeneity of variance.

A higher content of GLSs in biennial (winter rapeseed) compared to annual (spring rapeseed) cultivars, as seen in our study, was also found in a previous field study, where both types were grown in spring (Sarwar & Kirkegaard, 1998). Thus, Sarwar and Kirkegaard (1998) also found that the content of GLSs decreased as the plants grew bigger and came nearer to flowering, supporting our results with respect to dilution and effects of ontogeny, where flowering has been found to reduce the content of GLSs in rapeseed (Clossais‐Besnard & Larher, 1991). Furthermore, the biennial cultivars did not approach formation of buds due to lack of vernalization, which could explain the elevated GLS content compared to the annual cultivars in both years, because the biennial cultivars were kept at their vegetative stage, as argued by Sarwar and Kirkegaard (1998). Although no visible signs of bud formation were seen in the annual cultivars in 2017.

The content of bitter‐tasting GLSs (progoitrin, glucobrassicin, and neoglucobrassicin) at stage I differed among cultivars and years with “Silvershadow” containing the lowest amount of bitter‐tasting GLSs in both years (Table 1). In 2016, “Witt” had the highest content in contrast to “Jadak” in 2017. The content of bitter‐tasting GLSs was higher in 2017 compared to 2016 following the trend of glucobrassicin, which was the dominant bitter‐tasting GLS. Based on practical experience, the baby leaf salad of the white‐flowering rapeseed is thought to taste more mild and less bitter and astringent than baby leaf salad from the yellow‐flowering rapeseed. These differences might be linked to the content of the mentioned GLSs and their breakdown products (Mølmann et al., 2015; Pasini et al., 2011). However, we found no effects of flower color on bitter‐tasting GLS contents and GLS content in general (Table 1 to 3), which confirmed the results for seedlings of the same cultivars (Groenbaek et al., 2018b).

### Effect of cultivar, life cycle, and year on sensory profile

The results of the sensory evaluations in 2016 and 2017 are shown in Table 4. The results of the 2 years are not directly comparable, as the composition of the sensory panel and the cultivars evaluated differed between the 2 years. “Lysidé” was included in the evaluation in 2016, but not in 2017. As seen in Table 4, four of the evaluated sensory attributes were significantly different between the cultivars in 2016, whereas only bitterness differed between the cultivars in 2017. “Silvershadow” and “Lilput” had the lowest intensity of bitterness in 2017, whereas “Silvershadow” had the lowest intensity of bitterness in 2016, although the level was not significant. In 2016, the intensity of flower vase water aroma was higher for the annual cultivars compared with the biennial cultivars (Table 4). In contrast, biennial cultivars had a higher intensity of rapeseed aroma and bitterness. The same result for bitterness was found in 2017, whereas flower vase water aroma intensity was higher for the biennial cultivars. Moreover, fresh green aroma was found in higher intensities in the biennial cultivars compared with the annual cultivars. There were no differences in any of the sensory attributes when the group of white‐flowering rapeseed was compared with yellow‐flowering rapeseed (data not shown). The reason why bitterness only differed between the cultivars in 2017 could be that “Lysidé” was excluded from the sensory analysis in 2017 or that the content of GLSs was generally higher in 2017 than in 2016.

**Table 4:** Sensory quality of seven white‐ or yellow‐flowering, annual or biennial rapeseed cultivars harvested as baby leaves (stage I) in 2016 and 2017. Attributes were evaluated on a 15‐cm line scale

Year	Life cycle	Cultivar (cv)	Flower vase water aroma	Rapeseed aroma	Fresh green aroma	Horseradish aroma	Sourness	Bitterness	Rapeseed flavor	Pea pod flavor	Astringency
2016	Annual	Lysidé (w)^a^	6.1 bc^b^	6.4 b	8.7	7.6 a	6.6	7.3	7.3	6.8 abc	7.1
		Silvershadow (w)	10.0 a	6.7 b	8.9	3.7 b	6.0	5.6	7.8	7.4 abc	4.3
		Fenja (y)	8.8 ab	8.3 ab	9.1	8.4 a	6.9	6.8	8.8	8.6 a	6.2
	Biennial	Jadak (w)	5.5 c	8.6 ab	9.0	7.4 a	7.3	7.7	8.4	7.3 abc	6.3
		Witt (w)	7.7 abc	7.5 ab	9.0	6.2 ab	7.0	7.2	8.3	6.3 bc	6.7
		Lilput (w)	5.4 c	9.9 a	10.3	7.5 a	7.4	7.8	9.0	5.1 c	7.6
		Labrador (y)	6.3 bc	8.3 ab	9.6	7.2 a	6.4	7.1	7.1	7.5 ab	6.2
Cv effects			**^c^	*	NS	**	NS	NS	NS	***	NS
	Annual		8.2 a	7.1 b	8.9	6.6	6.5	6.6	7.9	7.6	5.9
	Biennial		6.2 b	8.6 a	9.5	7.1	7.1	7.5	8.2	6.5	6.7
Life cycle effect			**	*	NS	NS	NS	NS	NS	NS (0.0578)	NS
2017	Annual	Silvershadow (w)	7.3	9.0	7.6	7.6	8.6	7.9 b	9.0	7.0	6.6
		Fenja (y)	5.7	7.1	7.2	10.5	9.0	8.7 ab	7.9	6.1	8.7
	Biennial	Jadak (w)	9.5	6.2	9.6	7.3	7.4	9.3 ab	7.8	7.3	8.1
		Witt (w)	8.5	7.8	8.1	7.3	9.4	10.9 a	8.6	7.8	7.1
		Lilput (w)	8.6	7.3	8.2	9.4	6.2	7.6 b	9.3	7.6	6.6
		Labrador (y)	8.4	7.4	9.6	6.7	10.2	10.5 a	8.6	7.1	9.6
Cv effects			NS	NS	NS	NS	NS	*	NS	NS	NS
	Annual		6.5 b	8.0	7.4 b	9.1	8.8	8.3 b	8.4	6.5	7.6
	Biennial		8.7 a	7.2	8.9 a	7.7	8.3	9.6 a	8.6	7.4	7.8
Life cycle effects			*	NS	*	NS	NS	*	NS	*NS*	*NS*

a w, white flower color; y, yellow flower color.

^b^Different letters indicate significant differences among means (*n* = 3).

^c^NS, not significant; ^*^, *P* ≤ 0.05; ^**^, *P* < 0.01; ^***^, *P* < 0.001.

### The relation between sensory profile and content of glucosinolate

**Figure 4:**
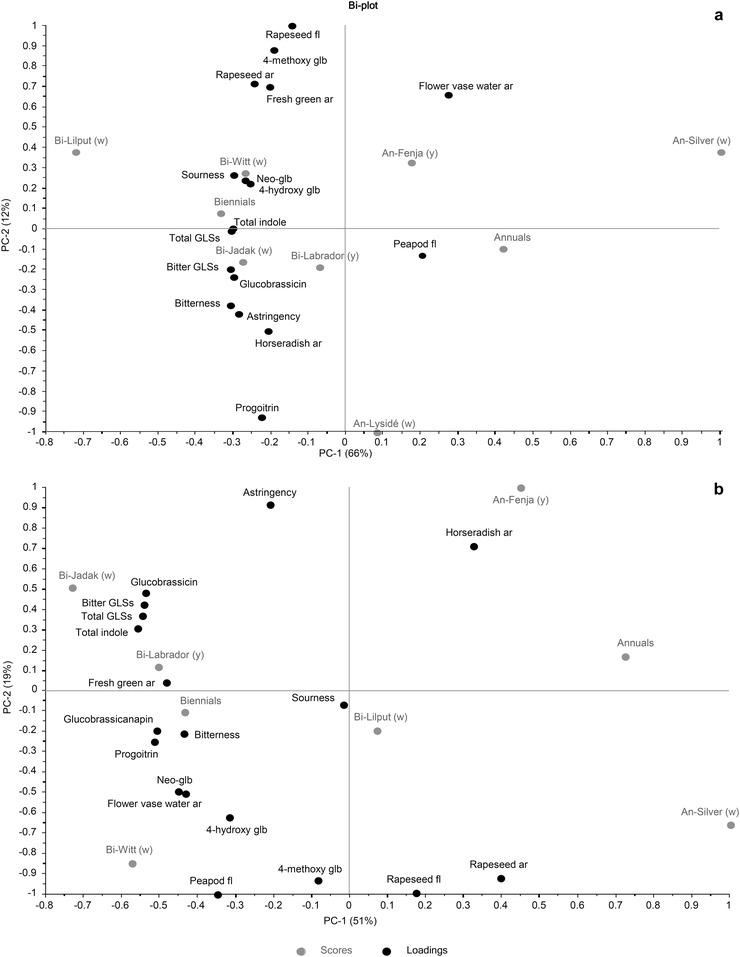
PCA biplot of sensory attributes and glucosinolates from seven white‐ or yellow‐flowering cultivars of rapeseed at the growing stage baby leaf, stage I in 2016 (a) and 2017 (b). An, annual; Bi, biennial; w, white flower color; y, yellow flower color; ar, aroma; fl, flavor; GLSs, glucosinolates; glb, glucobrassicin.

The sensory profile was found to be affected by the GLS content, as shown by the PCA biplots for the 2 years (2016 and 2017; Figure 4). Besides the individual GLSs, which were found to differ between cultivars or life cycle, the total content of the potential bitter‐tasting GLSs has been included along with the total content of indole and all GLSs.

As seen in Figure 4, the difference between life cycles for both years (PC1 66% and 51% for 2016 and 2017, respectively) had the greatest effect on the variation. The biennial cultivars were characterized by a high intensity of bitterness and astringency as well as a high content of individual bitter‐tasting GLSs and a total content of bitter‐tasting GLSs. The cultivars seemed to react differently the 2 years. Bitterness varied significantly in 2017, and the bitterness was highly related to both the content of bitter‐tasting GLSs as well as to total GLSs (Figure 4b). The same relationship between bitterness and content of bitter‐tasting GLSs and total GLSs is shown in Figure 4a, even though bitterness did not contribute to the variation, as it was not significant. The analysis confirms that the three individual GLSs included in the calculation of the content of bitter GLSs are the most bitter‐tasting GLSs as stated in previous studies (Drewnowski & Gomez‐Carneros, 2000; Engel et al., 2002; Mithen et al., 2000; Mølmann et al., 2015; Pasini et al., 2011; Schonhof et al., 2004; van Doorn et al., 1998). However, progoitrin seemed to show a weaker relationship with bitterness in 2016 (Figure 4a), just as 4‐hydroxy glucobrassicin and glucobrassicanapin (in 2017) may also have contributed to the bitterness.

The identification of bitter‐tasting GLSs from the above‐mentioned studies relies on a broad spectrum of experimental setups, including different species and processing methods. Previous studies have mainly revealed the relationship between bitterness and the aliphatic GLSs progoitrin, sinigrin, and glucosativin (Drewnowski & Gomez‐Carneros, 2000; Engel et al., 2002; Mithen et al., 2000; Pasini et al., 2011; Schonhof et al., 2004; van Doorn et al., 1998), whereas the results of the present study show that glucobrassicin was the main GLS contributor to the bitterness in the group of bitter‐tasting GLSs followed by progoitrin and neoglucobrassicin (Figure 4b). However, since the GLS profiles of the different vegetables differ, the results are not directly comparable. Our finding that these three GLSs were the major contributors to bitterness in baby leaf rapeseed consolidated the rather weak relationships found by Mølmann et al. (2015) and Engel et al. (2002).

Bell, Methven, Signore, Oruna‐Concha, and Wagstaff (2017) indicated that GLSs found only in low levels in a limited number of cultivars might result in distinct flavors of these specific cultivars. They found this for 4‐hydroxy glucobrassicin and glycoalyssin and their correlation with bitter aftereffects, pepper flavor, and mustard flavor and aftereffects amongst others. In the present study, glycoalyssin was not identified, and no differences in the content of 4‐hydroxy glucobrassicin were found among cultivars, which limits the support of our findings to the above‐mentioned. However, a higher content of 4‐hydroxy glucobrassicin found in the biennial cultivars and the relationship between 4‐hydroxy glucobrassicin and bitterness given from the PCA biplots indicated a contribution to the higher intensities of bitterness, although glucobrassicin was the main contributor among the indole GLSs (Table 1). Moreover, as glucobrassicanapin varied among cultivars and life cycles and showed a high relationship with bitterness in 2017, this GLS might contribute to bitter taste (Figure 4b).

## Conclusion

Life cycle was the main factor influencing the content of GLSs in baby leaf rapeseed with the biennial cultivars having the highest content. This influence also accounted for the bitter‐tasting GLSs and the sensory evaluation of bitterness. By shifting to the later sowing date and thereby harvest time the content of GLSs increased and the effect of repeated harvesting on GLS content and composition differed between seasons compared to undisturbed plant growth. As the total content of GLSs and bitter‐tasting GLSs corresponded well with the bitterness intensity, we conclude that life cycle and seasonal effects affected the sensory profile of baby leaf rapeseed. Thus, depending on consumers’ sensory preferences and the content of GLSs desired from a health perspective, growers should take life cycle and growing season into account by growing primarily biennial cultivars later, during a cooler season, if they wish to achieve a higher content of GLSs.

## Authors’ Contributions

M. Groenbaek secured the funding for the project, designed the field study, collected the field data, did the data analysis and interpretation, and drafted the manuscript.

E. Tybirk bred the tested cultivars, contributed to the field experiment with knowledge on plant growth and behavior, and contributed to the manuscript with facts and comments.

U. Kidmose conducted the sensory study, did the sensory data analysis, and contributed to the manuscript on sensory aspects.

H. L. Kristensen contributed to experiment planning, discussed the data analysis, results, and interpretation, and thoroughly commented on and contributed to the manuscript.

## Supporting information


**Table S1**. List of attributes and definitions of attributes
