# Supplementary material for: Glucosinolate Content and Sensory Evaluation of Baby Leaf Rapeseed from Annual and Biennial White‐ and Yellow‐Flowering Cultivars with Repeated Harvesting in Two Seasons
Source: J Food Sci. 2019 Jun 25;84(7):1888–99. doi: 10.1111/1750-3841.14680 (PMC6773201; doi:10.1111/1750-3841.14680)
Supplement: Supplementary file 1 — Table S1. List of attributes and definitions of attributes [file JFDS-84-1888-s001.docx]

**Supplementary Table 1S. List of attributes and definitions of attributes**

| **Group** | **Descriptor** | **Definition** |
| --- | --- | --- |
| Aroma^a^ | Flower vase water | Decay, rot, stored cut grass, old cabbage. |
|  | Rapeseed | Honey from a rapeseed field, flowering rapeseed field |
|  | Fresh green | Pea pod, spinach, newly cut grass, fresh beech leaves after squeezing the samples between the fingers |
|  | Horseradish | Acrid, mustard, radish after squeezing the samples between the fingers |
| Taste | Sourness | The basic taste of sourness. |
|  | Bitterness | The basic taste of bitterness. |
| Flavour | Rapeseed | Rapeseed field, rapeseed |
|  | Pea pod | Newly harvest pea pods, Grass, cucumber. |
| Sensation | Astringency | A drying-out sensation in the mouth |

^a^Aroma attributes are evaluated after shaking the beaker and removing the lid
